# Quantitative dual contrast photon-counting computed tomography for assessment of articular cartilage health

**DOI:** 10.1038/s41598-021-84800-x

**Published:** 2021-03-10

**Authors:** Petri Paakkari, Satu I. Inkinen, Miitu K. M. Honkanen, Mithilesh Prakash, Rubina Shaikh, Miika T. Nieminen, Mark W. Grinstaff, Janne T. A. Mäkelä, Juha Töyräs, Juuso T. J. Honkanen

**Affiliations:** 1 Department of Applied Physics, University of Eastern Finland, 70210 Kuopio, Finland; 2 Center of Oncology, Kuopio University Hospital, Kuopio, Finland; 3 Research Unit of Medical Imaging, Physics and Technology, University of Oulu, Oulu, Finland; 4 Diagnostic Imaging Center, Kuopio University Hospital, Kuopio, Finland; 5 A. I. Virtanen Institute for Molecular Sciences, University of Eastern Finland, Kuopio, Finland; 6 Medical Research Center, University of Oulu, Oulu, Finland; 7 Department of Diagnostic Radiology, Oulu University Hospital, Oulu, Finland; 8 Departments of Biomedical Engineering, Chemistry and Medicine, Boston University, Boston, USA; 9 School of Information Technology and Electrical Engineering, The University of Queensland, Brisbane, Australia; 10 Medical Imaging and Radiation Therapy, Kymenlaakso Central Hospital, Kymenlaakso Social and Health Services, Kotka, Finland

**Keywords:** Osteoarthritis, Imaging techniques, Cartilage, Computed tomography

## Abstract

Photon-counting detector computed tomography (PCD-CT) is a modern spectral imaging technique utilizing photon-counting detectors (PCDs). PCDs detect individual photons and classify them into fixed energy bins, thus enabling energy selective imaging, contrary to energy integrating detectors that detects and sums the total energy from all photons during acquisition. The structure and composition of the articular cartilage cannot be detected with native CT imaging but can be assessed using contrast-enhancement. Spectral imaging allows simultaneous decomposition of multiple contrast agents, which can be used to target and highlight discrete cartilage properties. Here we report, for the first time, the use of PCD-CT to quantify a cationic iodinated CA4+ (targeting proteoglycans) and a non-ionic gadolinium-based gadoteridol (reflecting water content) contrast agents inside human osteochondral tissue (*n* = 53). We performed PCD-CT scanning at diffusion equilibrium and compared the results against reference data of biomechanical and optical density measurements, and Mankin scoring. PCD-CT enables simultaneous quantification of the two contrast agent concentrations inside cartilage and the results correlate with the structural and functional reference parameters. With improved soft tissue contrast and assessment of proteoglycan and water contents, PCD-CT with the dual contrast agent method is of potential use for the detection and monitoring of osteoarthritis.

## Introduction

Articular cartilage is a highly specialized tissue that provides a smooth, lubricated, and elastic surface between the articulating bones. The tissue absorbs shocks and transmits loads to the underlying subchondral bone. This is made possible by the highly specialized composition of predominantly water, collagen fibers, and proteoglycans (PGs)^1,2^. Because of its avascular nature, articular cartilage exhibits a very limited capacity for regeneration. An acute joint injury, for example, in the form of articular fracture, meniscal tear, or ligament rupture, may initiate the development of post-traumatic osteoarthritis (PTOA)^3^. PTOA is a degenerative joint disease that typically leads to joint pain, stiffness, and reduced function^4^. The early changes associated with PTOA include loss of PGs, fibrillation and breakdown of collagen, and an increase in water content^5,6^. No disease curing drugs exist, but if the initiated degradation, i.e. decreased PG content and increased water content, could be detected early, e.g. while imaging an acute joint injury, the development of PTOA can be slowed down or even stopped with a pharmaceutical or surgical intervention^7–9^.

Contrast-enhanced computed tomography (CECT) has been investigated for the detection of PTOA related changes^10–12^. Contrast agents increase X-ray attenuation and, when taken-up by soft tissue, significantly improve the contrast of that tissue^13^. The diffusion of mobile contrast agent ions depends on the degenerative state of articular cartilage^14^. Current clinically available contrast agents are anionic or non-ionic, and thus, are not attracted to articular cartilage which possesses an overall negative fixed charge density due to the PGs. Therefore, the PG distribution cannot be quantified directly. To overcome this restriction, a cationic contrast agent was introduced and it shows superior sensitivity to detect PG content at diffusion equilibrium^15–17^. However, other degeneration related factors affect the diffusion and reduce the sensitivity before equilibrium has been established. As the PG content decreases, the uptake of the cationic molecules reduces. However, the diffusion is simultaneously accelerated due to the increased porosity, water content, and increased surface area due to fibrillation. For this reason, the use of cationic contrast agents has been limited in detecting structural degeneration at early time points after the administration.

To enhance the capability of CECT-imaging, we developed a quantitative dual-energy CT (QDECT) technique that uses a mixture of non-ionic gadolinium-based gadoteridol and cationic iodinated CA4+ contrast agents^18–21^. The use of QDECT with the two contrast agents allows quantitative assessment of two important health reflecting properties of the articular cartilage, i.e., water and PG content. The diffusion of the non-ionic gadoteridol is governed only by cartilage water content and steric hindrance. Hence, the ability to normalize the effect of these attributes on the diffusion of the CA4+ should improve the sensitivity to assess the cartilage properties. The presented imaging technique could be used, for example, in the case of an acute joint trauma. Further, this technique could aid the physician to quantitatively evaluate the severity of the injury and select a suitable treatment.

Spectral information is needed to estimate the concentrations of two contrast agents^22^, and the optimal selection of the X-ray energies depends on the absorption edges of the quantified contrast agents. With current clinical DECT devices, spectral information is acquired with two different X-ray spectra. Clinical DECT devices use energy-integrating detectors (EIDs) which convert the X-ray photons to light in the scintillator material and the light is then detected with photodiodes. Thus, these detectors detect only the incoming photons but cannot provide specific information about the photons’ energy. Therefore, to get the spectral information for the concentration estimation of two contrast agents, the sample needs to be scanned with two different spectra, for example, using fast kVp-switching^23^ or dual-source CT^24^ methods.

The spectral discrimination with photon-counting detectors (PCDs) is fundamentally different to that of EIDs. The X-ray photons are converted to a charge cloud in the conversion medium, e.g. CdTe or CdZnTe, and the bias voltage propagates the charge cloud to the pixelated anode. Because the magnitude of the charge cloud is directly proportional to the energy of the converted photon, the photon energy can be analyzed with a pulse height discriminator. Photons are then classified into energy bins, thus enabling energy selective imaging within the single acquisition. Since PCDs have higher detection efficiency than EIDs^25,26^, PCD-CTs can enable the use of a lower dose and similar or improved image quality with similar noise levels compared to the conventional CT scanners^25,26^. However, if the spectral information is applied for quantitative imaging through the material decomposition process, the noise is amplified in the material basis images^27^.

Thus far, PCD-CT has been successful in quantifying iodine and gadolinium dual contrast agents in colon^28^, liver^29,30^, and heart^31^. Recently, a full-body contrast-enhanced PCD-CT was applied in the imaging of monoiodoacetate degraded articular cartilage in a porcine model^32^. In this study, gadolinium was injected into joint space and gadolinium concentration maps were determined. The spectral data also allowed to estimate the subchondral bone density based on hydroxyapatite phantoms. The suitability of PCD-CT with non-ionic gadolinium-based gadoteridol and cationic iodinated CA4+ contrast agents has not yet been investigated for the QDECT technique.

In this study, we apply photon-counting detector computed tomography (PCD-CT) to the previously introduced dual contrast agent method^18–21^. We employ the two bin PCD-CT system with dual contrast agent method to study human articular cartilage. Furthermore, we are evaluating the capability of the experimental PCD-CT setup to assess the biomechanical and histological properties of cartilage. Our goals are to (1) establish a protocol for PCD-CT setup, (2) validate the PCD-CT protocol for the QDECT technique, and (3) investigate the capability of PCD-CT to detect degeneration differences in human articular cartilage.

## Materials and methods

### Osteochondral samples

**Figure 1:**
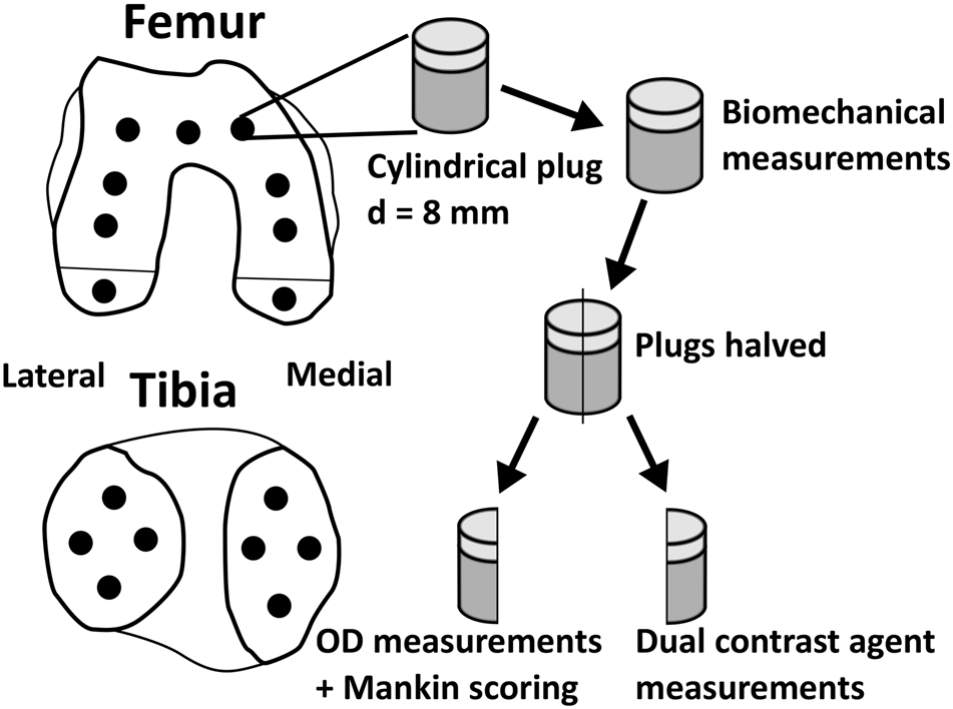
Osteochondral plugs (*n* = 57, *d* = 8 mm) were extracted from two human cadavers (aged 68 and 79 years). Four samples were excluded from the original sample pool due to missing cartilage, leaving 53 samples for the final analysis. First, the biomechanical properties were determined. Then the plugs were halved, and one half was used for optical density (OD) measurements and Mankin score grading. The other half was used for dual contrast agent measurements.

Osteochondral samples (*n* = 57 cylinders, *d* = 8 mm) were collected from two human cadaver knee joints (aged 68 and 79 years, Fig. 1). Coring was done under water-irrigation using a cylindrical coring drill attached to a drill press. The samples were collected from the load-bearing surfaces of femoral condyles and tibial plateaus and preserved frozen (− 20 °C) while soaked in PBS (phosphate-buffered saline, VWR International, Radnor, PA, USA) supplemented with protease inhibitors [EDTA (ethylenediaminetetra-acetic acid disodium salt dihydrate, *C* = 1.86 g/L, VWR International, Radnor, PA, USA) and benzamidine hydrochloride hydrate (*C* = 0.78 g/L, Sigma-Aldrich Co., St. Louis, MO, USA)]. The human tissue collection was approved by the Research Ethics Committee of the Northern Savo Hospital District, Kuopio University Hospital (decision numbers 58/2013 and 134/2015) and all the methods fulfilled all the relevant guidelines and legislations.

### Biomechanical indentation

Equilibrium and instantaneous moduli were determined for the samples in indentation geometry using a custom-made testing system^18,19,21,33^. A stress-relaxation protocol was implemented using a ramp strain rate of 100% per second with three compressive steps. Each step was 5% of the remaining uncompressed cartilage thickness with a relaxation period of 15 min after each step^34^. Equilibrium modulus was calculated as a linear fit of the stress–strain ratio at equilibrium points. Instantaneous modulus was calculated from the ramp phase of the third step. The diameter of the indenter was 728 µm for one cadaver and 667 µm for the other, due to a breakage of the first indenter. The moduli were calculated using the solution derived by Hayes et al.^35^ which takes into account the changed indenter radius and the finite thickness of cartilage. Poisson’s ratios used in the calculations were 0.2 and 0.5 for the equilibrium and instantaneous modulus, respectively^36^. More details are presented in the Supplementary Material.

### Handling of the samples and contrast agent solutions

After the biomechanical measurements, the cylindrical samples were halved; one half cylinder was used for the contrast agent measurements and the other half for histological grading (Fig. 1). Four samples were excluded from the original sample pool due to missing cartilage, leaving 53 samples for the analysis. Sides of the CT imaged samples were sealed using cyanoacrylate (Loctite, Henkel Norden AB, Dusseldorf, Germany) to allow the contrast agent diffusion only through the articulating surface. The same samples were utilized in an earlier study by Bhattarai et al.^18^ and had been infused with iodinated and gadolinium-based contrast agents for 72 h to reach diffusion equilibrium^37^. The samples were frozen second time in individual plastic bags at − 20 °C before the PCD-CT scanning. The dual contrast agent bath was comprised of cationic iodinated CA4 + (*q* =  + 4, *M* = 1499.9 g/mol) and non-ionic gadolinium-based gadoteridol (Prohance, Bracco International B. V., Amsterdam, Netherlands; *q* = 0, *M* = 558.7 g/mol). The concentration of the contrast agents in the bath was 24 mg·I/mL and 24 mg·Gd/mL for CA4+ and gadoteridol, respectively. The osmolality of the solution was adjusted to 308 m·Osm/kg with sodium chloride and determined using a freezing point osmometer (Halbmikro-osmometer, GWB, KNAUER Wissenschaftliche Geräte GmbH, Berlin, Germany).

### Optical density

**Figure 2:**
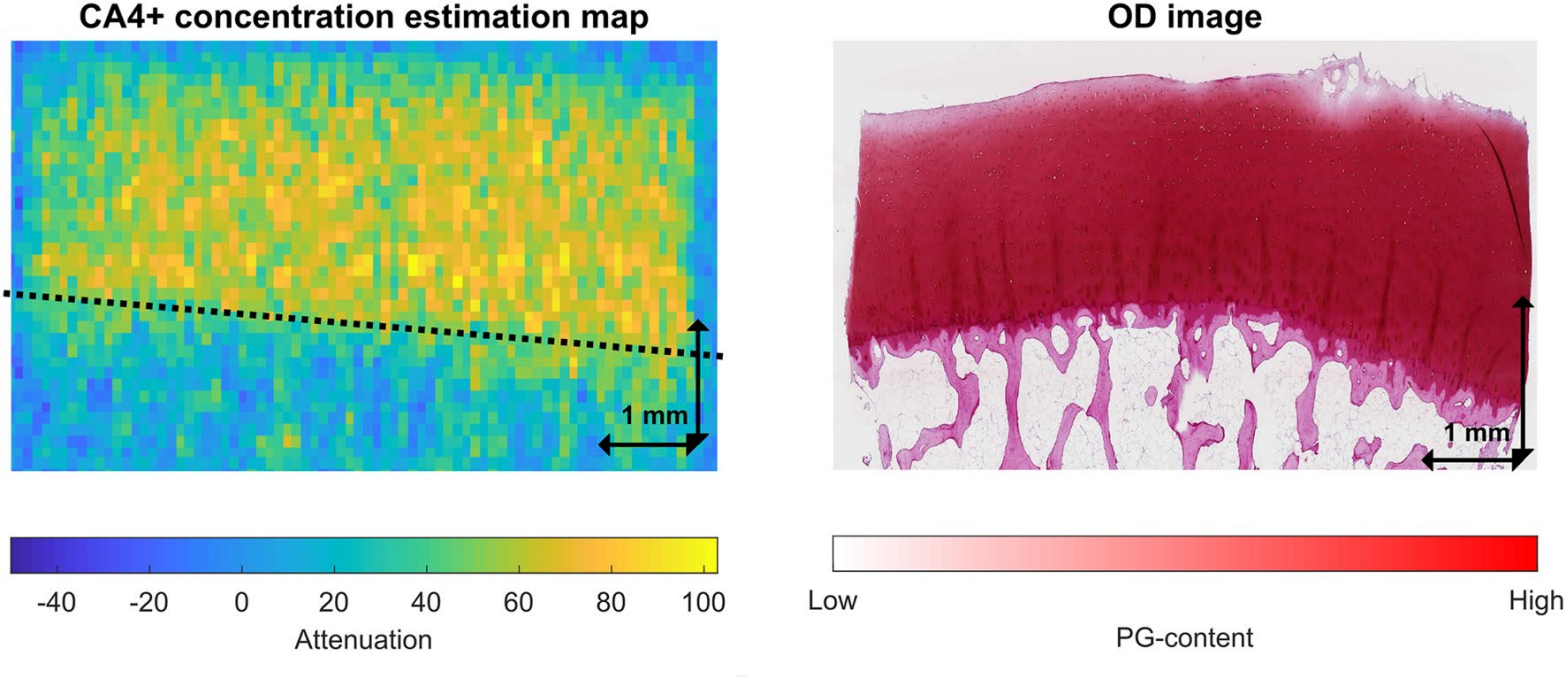
CA4+ concentration map (left) and optical density (OD) image (right) of a single representative sample. The dotted black line indicates roughly where the bone-cartilage borderline is. The articular cartilage surface is at the top part of the image and the subchondral bone can be seen at the bottom.

The second halves of the samples were fixed in 10% formalin, decalcified in EDTA, processed in graded alcohol solutions, and embedded in paraffin. Subsequently, the samples were cut into 3 µm thick sections and stained with Safranin-O^19^. Safranin-O is a stain that binds stoichiometrically with matrix PG. Spatial PG content was determined from the optical density (OD) of the sections, measured using digital densitometry (Fig. 2). The measurement system consisted of a light microscope (Nikon Microphot-FXA, Nikon CO., Tokyo, Japan) with a monochromatic light source (*λ* = 492 ± 8 nm), Plan 1/0.04 objective (Carl Zeiss AG, Oberkochen, Germany), and a 12-bit CCD camera (ORCA-ER, Hamamatsu Photonics K.K., Hamamatsu City, Japan)^38^. The calibration was conducted using neutral density filters ranging from 0 to 3.0. Transversely averaged depthwise profiles were created at 1% increments of normalized depth. The OD for each sample was determined as the average of three sections.

### Histological grading

Mankin histological grading system was used to evaluate the severity of osteoarthritis (OA)^39^. The scoring was done using the same sections used for the digital densitometry. The sections were blind-coded and graded by four independent assessors. Each sample had three sections, which were graded. The grading was carried out with a combined score evaluating structure (0–6 points), cellular abnormalities (0–3 points), matrix staining (0–4 points), and tidemark integrity (0–1 point). The final score of the section was the average of the grade of the three sections.

### PCD-CT setup

**Figure 3:**
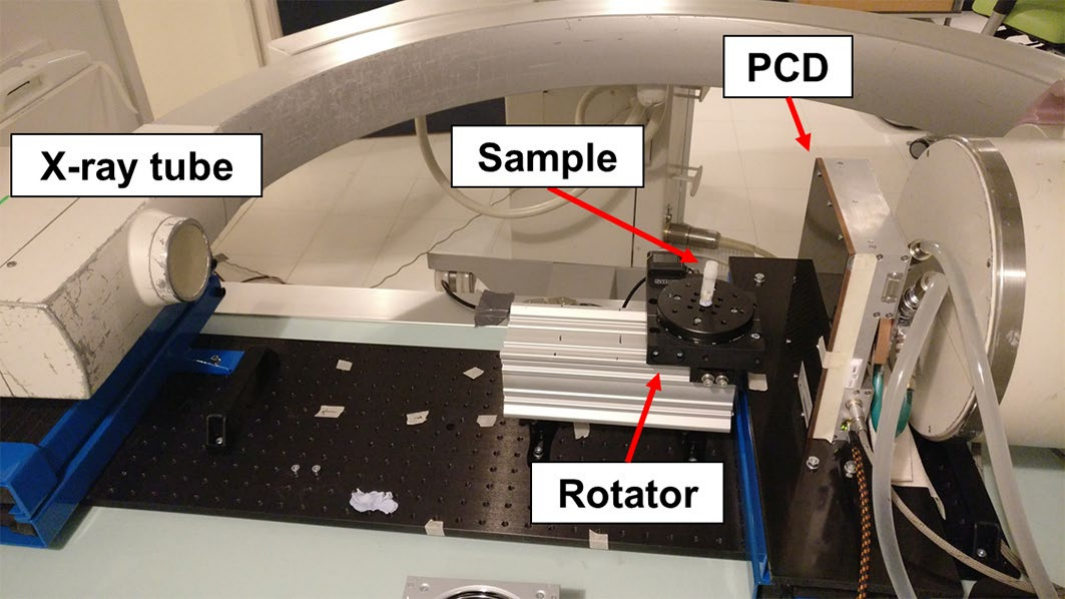
Image of the experimental photon-counting CT setup used in the current study. Photon-counting detector (PCD) is in front of the C-arm’s own detector that is not used in this setup.

The experimental spectral PCD-CT setup consisted of Xflite PCD (XC-Flite FX15, XCounter AB, Danderyd, Sweden), a motorized rotator (NR360S, Thorlabs Inc., NJ, USA), and C-arm X-ray source (BV 29, Philips, Amsterdam, Holland) (Fig. 3). Xflite PCD has 0.75 mm thick cadmium telluride as energy conversion material. PCD was mounted on a scissor jack, and a motorized rotator was positioned on an optical breadboard placed on a bucky table. The X-ray tube voltage was set to 100 kVp and the tube current to 3 mA. The sample was rotated with a speed of 6° per second  and the detector captured 1500 frames with a speed of 24 frames per second, leading to total rotation of 375°. This ensured that the sample was imaged completely. In the analysis phase, the frames beyond 360° were excluded, and two subsequent frames were averaged yielding 720 projections for a full rotation.

The active area of the detector panel was 51.3 × 154.7 mm^2^ divided into two rows of 12 tiles. It had two 12-bit counters per pixel with a pixel size of 100 × 100 µm^2^ and two adjustable energy thresholds set to 30 and 60 keV. The lower threshold determines the photons counted in the total energy (TE) bin and the higher threshold determines the photons counted in the high energy (HE) bin. TE and HE images were acquired during imaging and the low energy (LE) images were calculated offline as LE = TE − HE. The LE and HE bins were chosen based on the *K*-edges of iodine (33.2 keV) and gadolinium (50.2 keV) and on energy response of the PCD-CT system, which shows high-energy drift for photons over 40 keV^40^. Therefore, the upper threshold was set at 60 keV. Source to object distance was set to 77.60 cm, and object to detector distance of 11.58 cm was selected so that the rotator was as close as possible to the detector, resulting in a magnification of approximately 1.15 and a voxel size of 87.0 × 87.0 × 87.0 µm^3^ in the reconstructed volume. The spatial resolution of the imaging system was estimated by measuring the modulation transfer function (MTF). More detailed information about the MTF analysis can be found in the Supplementary Material. The MTF50% and MTF10% values for the PCD-CT system were 2.43 1/mm and 4.27 1/mm, respectively (Supplementary Fig. S3).

### Imaging protocol

The imaging protocol was as follows. First, projection images of the background, water, calibration, and validation solutions were acquired. The concentrations in the calibration solutions were 0, 8, 16, 24, 32, 40, and 48 mg·I/mL and mg·Gd/mL of CA4+ and gadoteridol, respectively. The validation mixture concentrations were 9.6/38.4, 19.2/28.8, 28.8/19.2, and 38.4/9.6 mg·(I/Gd)/mL. The contrast agent mixtures had been stored frozen and were thawed at room temperature. To avoid drying, the samples were thawed in their respective contrast agent mixtures. Imaging was conducted in air, one sample at a time. Samples were wiped with lint-free wipes and placed inside a sealed plastic holder containing a piece of wet paper towel to prevent dehydration during the scan.

### Preprocessing and reconstruction

**Figure 4:**
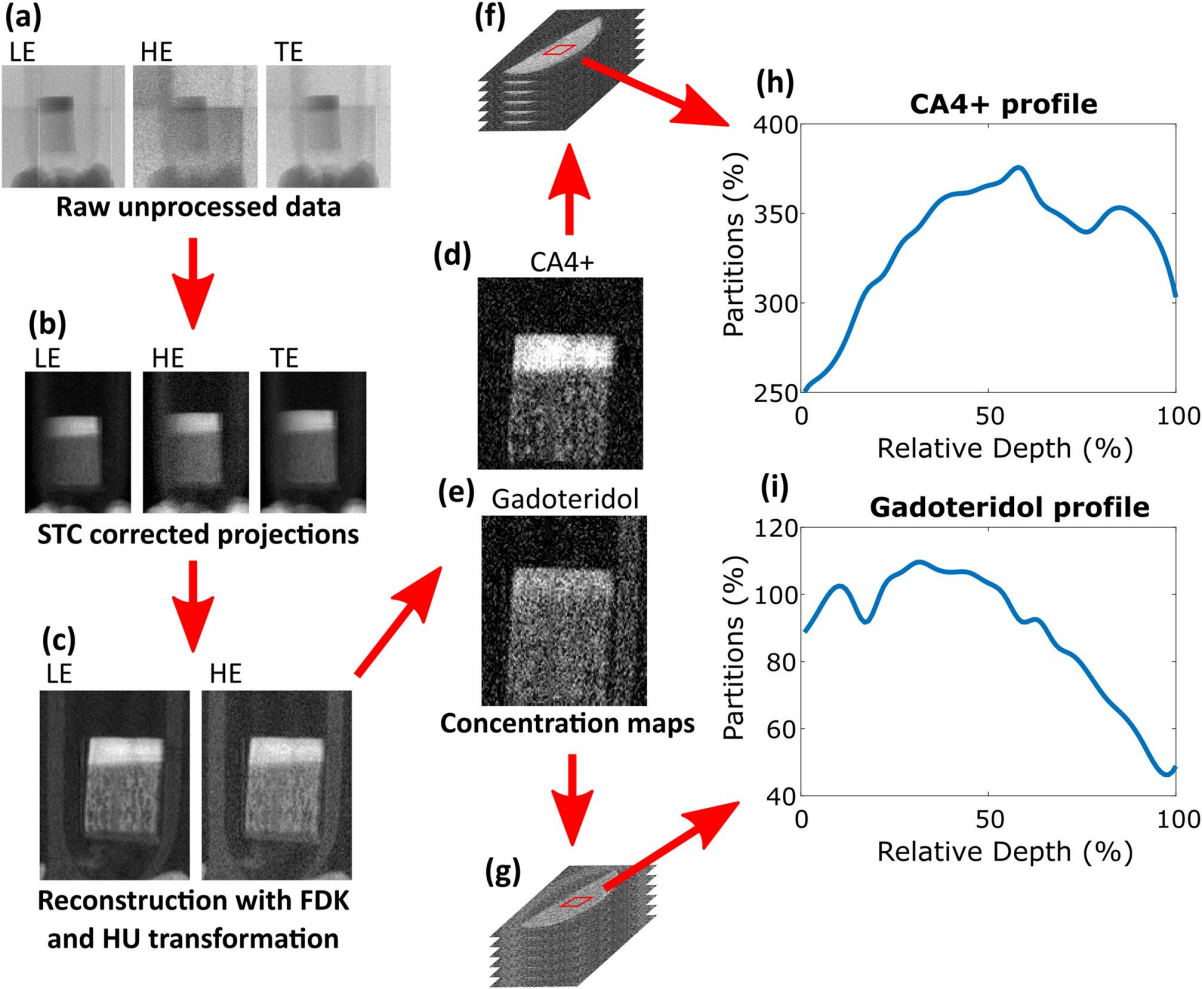
Representation of the analysis process. Raw data (**a**) was acquired using two energy bins: high energy (HE) and total energy (TE). Low energy (LE) bin data was calculated from the difference between the TE and HE data. The PCD’s tile gaps can also be seen in the raw data. Raw images were preprocessed using the signal-to-equivalent thickness calibration (STC) and projection images were created (**b**). The projection data were in turn reconstructed with the Feldkamp-Davis-Kress (FDK) algorithm and converted to Hounsfield units (HUs) (**c**). This allowed the contrast agent concentration maps (**d**, **e**) to be calculated. Volume of interest (red squares in subfigures **f**, **g**) was used to determine depth-dependent mean profiles (**h**, **i**) for the contrast agent partitions. 0% marks the cartilage surface and 100% the cartilage-bone interface.

Detailed information about preprocessing and reconstruction steps can be found in the Supplementary Material and are explained here shortly. The panel detector is constructed from tiles that have a 100 µm wide gap between them. These gaps appear in the raw images (Fig. 4a) and therefore tile gap interpolation was performed to smooth out tile edges. Subsequently, these images were then preprocessed using the signal-to-equivalent thickness calibration (STC)^41^ and a ring artifact removal algorithm^42^. The former was used to correct beam hardening and the tilewise variations (Fig. 4b)^41^.

3D reconstruction of each energy bin was calculated with the ASTRA Toolbox (toolbox for MATLAB, ver. 1.8) using the Feldkamp-Davis-Kress (FDK) method^43^ (Fig. 4c). All data analysis was done using MATLAB (R2018b, MathWorks, Natick, MA, USA). Only the LE and HE reconstruction images were used in the concentration estimation analysis to create the concentration maps (Fig. 4d, e).

### Concentration estimation for CA4+ and gadoteridol

The concentration estimation for CA4 + and gadoteridol was done by using calibration-based method^18,19^. A detailed description of the calibration can be found in the Supplementary Material. The contrast agent concentrations were transformed into contrast agent partitions by dividing the measured concentration with that of the surrounding contrast agent bath. The depthwise profiles (Fig. 4h,i) were formed by taking 20 × 20 pixels (approximately 1.74 × 1.74 mm^2^) square region-of-interests (ROIs) through the cartilage in the depth direction (Fig. 4f,g) and calculating a mean value for each ROI.

### Statistical analysis

Due to non-normal distributions of the reference parameters (Kolmogorov–Smirnov test, *p* < 0.0001), non-parametric tests were used. Spearman’s correlation coefficient *ρ* and significance *p*-values were calculated between the contrast agent partitions and reference data. The statistical significance limit was set to *p* < 0.05. Statistical analyses were performed with MATLAB (R2018b) using Statistics and Machine Learning Toolbox (Version 11.4). Bland–Altman plots for the validation solutions were calculated using MATLAB function by Klein^44^.

## Results

The attenuation values reflected reliably the contrast agent concentrations used for the calibration, *R*^2^ = 0.997 and *R*^2^ = 0.998 for CA4+ and gadoteridol, respectively (Supplementary Figs. S1a and S1c). The mean errors of the measured calibration values were 6% and − 4% for CA4+ and gadoteridol, respectively.

**Figure 5:**
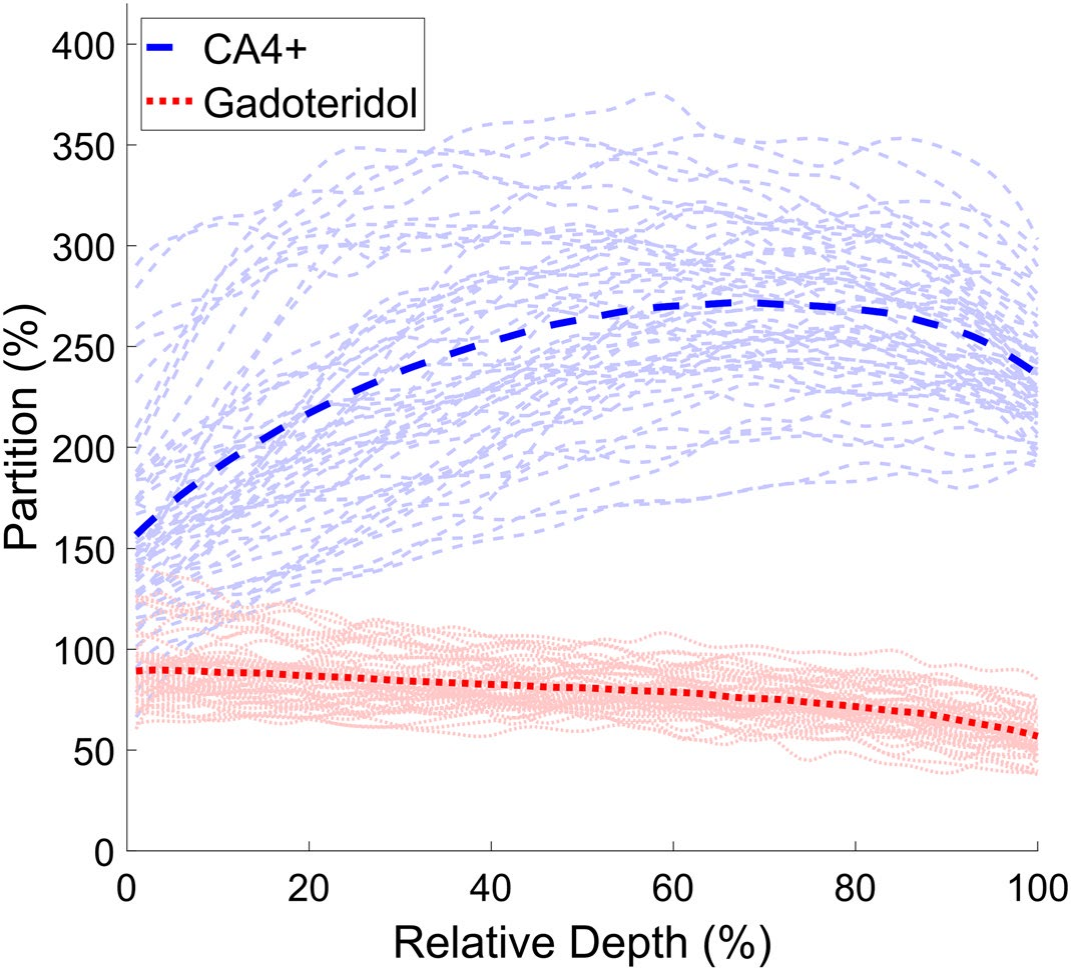
Depthwise partition profiles of CA4+ and gadoteridol. Lighter lines represent individual samples and darker lines the average profiles over all samples. The partition is calculated by dividing the estimated concentration of the contrast agent in cartilage with the starting concentration of the same contrast agent in the bath. The cartilage surface is at 0% and the cartilage-bone interface at 100%.

Average bulk (i.e., full thickness cartilage) contrast agent partition inside the samples was 241.3 ± 41.2% (range = 157–272%) for CA4+ and 78.6 ± 9.7% (range = 57–90%) for gadoteridol. The depthwise contrast agent partitions for CA4+ and gadoteridol are presented in Fig. 5. Average Mankin score for the samples was 6.22 ± 1.96 (range = 2.25–10.67). The average thickness of the cartilage was 2.86 ± 0.62 mm (range = 1.81–5.45).

**Figure 6:**
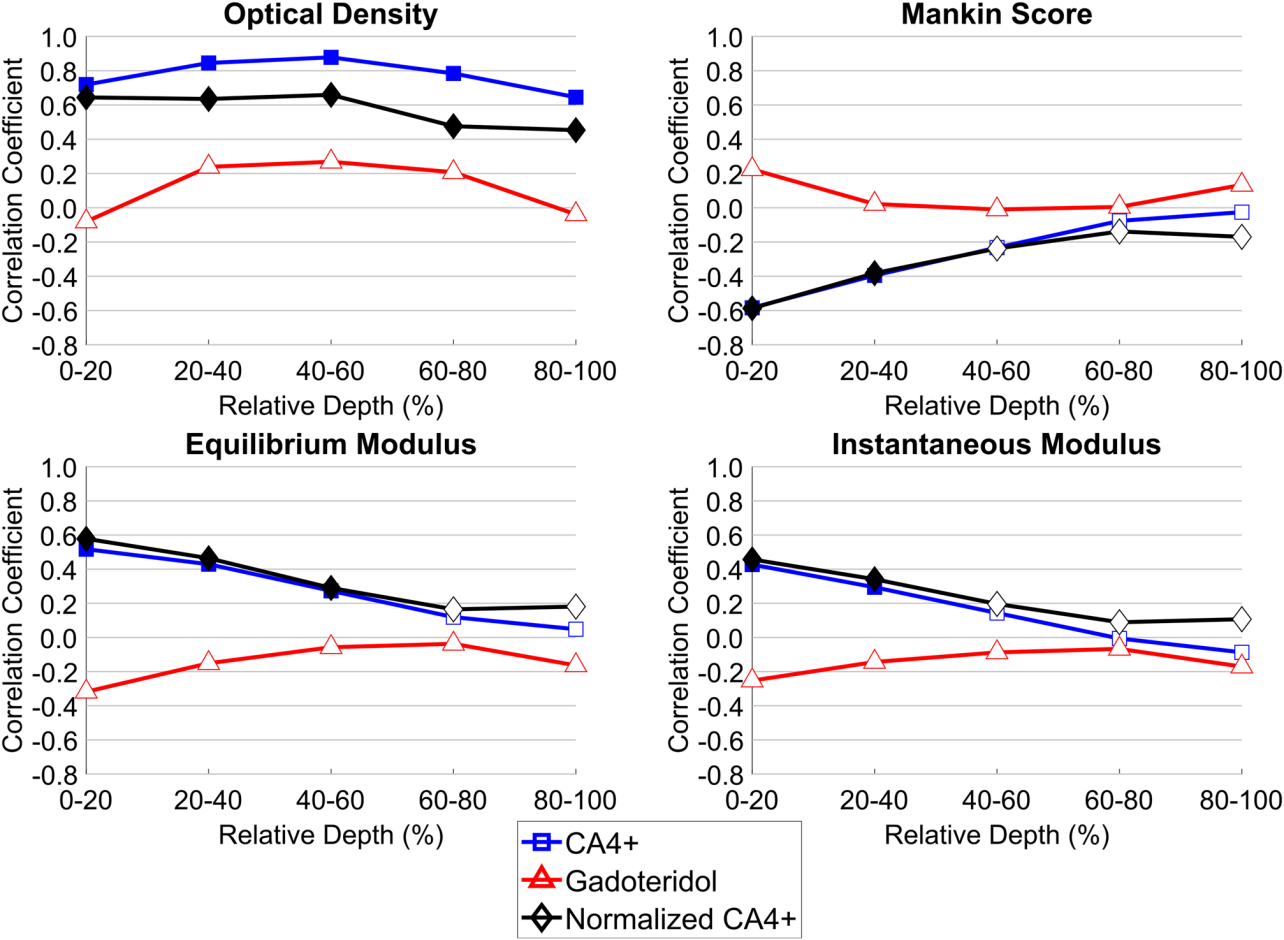
Depthwise Spearman’s correlation coefficients (*ρ*) between the reference parameters, and the CA4+ partition, normalized CA4+ partition and gadoteridol partition. Gadoteridol partition is provided for illustration purposes since it is used for the normalized CA4+. Filled marker indicates statistically significant correlation (*p* < 0.05). The cartilage surface is at 0% and the cartilage-bone interface at 100%.

Correlation coefficients between imaging and structural and functional parameters varied between different cartilage layers (Fig. 6). Correlation between CA4+ partition and OD was statistically significant in superficial zone (relative depth 0–20% of thickness) and bulk cartilage, *ρ* = 0.719, *p* < 0.0001 and *ρ* = 0.836, *p* < 0.0001, respectively. Bulk CA4+ partition correlated significantly with equilibrium modulus (*ρ* = 0.328, *p* = 0.0171). Correlation was also significant between the superficial CA4+ partition and the instantaneous and equilibrium moduli *ρ* = 0.427, *p* = 0.0015 and *ρ* = 0.517, *p* < 0.0001, respectively. Bulk CA4+ and superficial cartilage CA4+ partitions correlated with Mankin score, *ρ* = − 0.307, *p* = 0.0262 and *ρ* = − 0.584, *p* < 0.0001, respectively.

Normalization of CA4+ partition with that of gadoteridol increased the correlation coefficients between CA4+ partition and biomechanical properties (Table 1 and Fig. 6). Correlation between the CA4+ partition and OD was stronger without the normalization. Correlation between CA4+ partition and OD (Fig. 6) was strongest in the middle layer (relative depth of 40–60%). Normalized CA4+ partition had a strong correlation with OD in the superficial and middle cartilage (relative depth of 0–60%) and moderate correlation in the deep cartilage (relative depth of 60–100%).

**Table 1:** Spearman’s correlation coefficients (*ρ*) between the contrast agent partitions and reference data in bulk cartilage.

	CA4+	Gadoteridol	Normalized CA4+
PG-content^a^ (OD)	0.836***	0.205	0.637***
Instantaneous modulus	0.197	− 0.226	0.292*
Equilibrium modulus	0.328*	− 0.229	0.422**
Mankin score	− 0.307*	0.123	− 0.368**

^a^Optical density (OD) represents proteoglycan (PG) content of the cartilage.

Statistical significance: *p < 0.05; **p < 0.01; ***p < 0.001.

## Discussion

PCD-CT represents the latest technology in computed tomography, and CECT is an emerging imaging technique for quantitative, non-destructive, imaging of tissues, including but not limited to articular cartilage. The combination of PCD-CT and CECT allows efficient and quantitative assessment of the key cartilage quality and properties, such as PG and water content, as well as detection of early PTOA. This is the first study to combine these two methods with human articular cartilage and the results obtained are in agreement with the previous studies using conventional EIDs^18–21^.

The measured contrast agent partition profiles of the CA4+ and the gadoteridol (Fig. 5) resemble the physiological PG and water distributions of human articular cartilage^45^. The CA4+ partition positively correlates with the OD and biomechanical properties, i.e., instantaneous and equilibrium moduli, of articular cartilage. The PGs in cartilage forms the fixed charge density, which attracts the cationic CA4+ molecules, and is the rationale behind the strong positive correlation between CA4+ partition and OD. The amount of PGs is also directly proportional to the stiffness of the cartilage since they indirectly modulate the stiffness through osmotic pressure^46,47^. The correlation between the CA4+ and PG content agreed with the earlier results acquired using µCT^16^ and synchrotron µCT^21^. The correlations between CA4+ and biomechanical properties were also similar to earlier studies using µCT^18^ and synchrotron µCT^21^.

The spatial resolution of the PCD-CT is high enough to reveal the depth-dependent trend of the contrast agent partition. With clinical full-body CT, the reported correlations between CA4+ and OD or normalized CA4+ and OD were weaker compared to PCD-CT^19^. We expect that this poorer performance of clinical CT is due to larger slice thickness (0.5 mm) and the consequent partial volume effect. The experimental PCD-CT setup enables depthwise imaging and analysis with an isotropic voxel size of 87.0 × 87.0 × 87.0 µm^3^. The depthwise analysis shows a significant correlation between CA4+ partition and OD throughout the cartilage thickness (Fig. 6). This observation is similar to that previously reported by Honkanen et al. using full-body CT^19^ and synchrotron μCT^21^. The highest correlation between CA4+ partition and biomechanical parameters occurs in the superficial layer. This result was expected since biomechanical indentation testing, at an approximately 15% strain, reflects the properties of the superficial cartilage^48,49^.

The average Mankin score of the samples is 6.22 ± 1.96, representing a condition between healthy cartilage (Mankin = 0) and severe OA (Mankin = 14). Since the samples were gathered only from two cadavers, the variation in the cartilage’s condition is relatively small between the samples. Even though Mankin score grading provides the degenerative status of the whole cartilage, it tends to reflect more the superficial attributes of the tissue^50^, such as surface irregularities. Thus, the degenerated status of the samples, together with the characteristics of the Mankin scoring, likely explains the depthwise decrease in the significance of the correlation between the CA4+ partition and the Mankin score. Again, this finding is similar to the results previously reported by Honkanen et al.^19^.

The normalization of the CA4+ partition with the gadoteridol partition does not improve the correlation with the OD, biomechanical properties and Mankin scoring when discrete cartilage layers are inspected (Fig. 6). Yet, in bulk cartilage, the normalization slightly increases the correlation between the CA4+ partition and the biomechanical moduli, and Mankin score (Table 1). When small slices consisting of few voxels are inspected, local variance likely affected the power. Furthermore, at the diffusion equilibrium, the distribution of CA4+ should follow the exact PG distribution. Thus, at equilibrium, the normalization is the least effective.

To minimize the effect of beam hardening, we used STC with aluminum plates. STC provides better image quality with less noise than conventional flat field correction^40,41^. This is due to the increased fraction of photoelectric absorption of aluminum, which makes it relatively more comparable to the attenuation properties of iodine and gadolinium. Nonetheless, gadoteridol partitions surpass 100% in the samples, which theoretically should not happen. We suspect that the STC calibration with aluminum did not sufficiently correct for the beam hardening, especially at the high CA4+ concentrations, which results in erroneous gadoteridol concentration estimations. In a study by Honkanen et al.^21^, gadoteridol partitions surpass 100%, even though a monochromatic X-ray source was used, and the reason for the high gadoteridol concentrations was not found.

The calibration-based attenuation versus concentration curve is accurate, which was confirmed with validation measurements involving known mixture of CA4+ and gadoteridol (Supplementary Fig. S1). However, the validation also shows that the CA4+ concentration is overestimated approximately by 6% (Supplementary Fig. S1b) and gadoteridol concentration is underestimated approximately by 4% (Supplementary Fig. S1d). At high CA4+ concentrations (72 mg·I/mL around 300% partition), we observe a slight saturation in the calibration curve, which is most likely due to beam hardening. Therefore, solutions over 48 mg·I/mL were not selected or used in the calibration. This result is a limitation since the calibration solutions do not cover the range of contrast agent concentrations measured in the articular cartilage, and even the mean concentration of CA4+ in the present samples is high as about 58 mg·I/mL because the uptake of cationic ions into cartilage is more than 100%. This limitation might cause some errors in the estimation of higher CA4+ concentrations.

Another technical limitation is the relatively large focal spot (0.6 mm) of the X-ray tube of the used PCD-CT system. This limited the capability to increase the magnification of the PCD-CT system, since if the magnification is increased, the penumbra effect becomes significant in the images, interfering with the measured attenuation values and causing an error to the concentration estimation. Albeit, based on the MTF analysis the resolution of the PCD-CT system (0.234 mm, estimated from MTF10%) is comparable to a clinical full-body CT scanner^51^ and sufficient for the analyses conducted in this study.

In the future, we will explore material decomposition methods in projection^52^ or image space^53^, by utilizing the knowledge of the detector response and energy-dependent mass attenuation coefficients of iodine and gadolinium. This will allow quantitative analysis without a need for the calibration step used in this work since the attenuation coefficients are obtained from tables based on the effective energies of the energy bins. Such techniques have so far been successfully implemented by Muenzel et al.^29^ and Symons et al.^31^, for PCD-CT. We applied a three-material decomposition method to one of the samples, as a pilot study, to test the concentration estimation at deep cartilage, near the subchondral bone, to confirm the accuracy of the method. More information about the results of this study can be found in the Supplementary Material.

The present proof-of-concept study focuses on the validation of the custom PCD-CT experiment setup to be used for the dual contrast agent method. This method has not yet been applied to PCD-CT imaging of human articular cartilage. We acknowledge that the contrast agents and concentrations utilized in this study have some limitations from a clinical translational perspective, as such. Fortunately, the applied dual contrast method is not limited to the contrast agents used in this study, but other suitable contrast agents can be exploited as well, as long as notable differences in the spectral attenuation profiles (i.e. different *K*-edges) exists between the used contrast agents. Alternatively, the method allows 3D histology for in vitro samples without the need for sample preparation. The clinical applicability, protocol, and the effectiveness at earlier diffusion time points before the diffusion equilibrium is reached will be evaluated later. The results show that the method quantifies discrete contrast agent concentrations in human knee articular cartilage, and hence reflect the biochemical and biomechanical state of the tissue. Thus, PCD-CT imaging allows the detection of the initial degenerative changes in the articular cartilage, which enables early pharmaceutical or surgical interventions, when the initial OA is still treatable and not yet fully developed.

## Supplementary Information

Supplementary Information

## Data Availability

The datasets generated and analyzed during the current study are available from the corresponding author on reasonable request.
