# Supplementary material for: Quantitative dual contrast photon-counting computed tomography for assessment of articular cartilage health
Source: Sci Rep. 2021 Mar 10;11:5556. doi: 10.1038/s41598-021-84800-x (PMC7946949; doi:10.1038/s41598-021-84800-x)
Supplement: Supplementary file 1 — Supplementary Information [file 41598_2021_84800_MOESM1_ESM.docx]

**Supplementary Material 1**

For the manuscript:

**Quantitative Dual Contrast Photon-Counting Computed Tomography for Assessment of Articular Cartilage Health**

Petri Paakkari, Satu I. Inkinen, Miitu K. M. Honkanen, Mithilesh Prakash, Rubina Shaikh, Miika T. Nieminen, Mark W. Grinstaff, Janne T. A. Mäkelä, Juha Töyräs, and Juuso T. J. Honkanen

**Materials and Methods**

*Biomechanical Indentation*

Equilibrium and instantaneous moduli were determined for the samples. First, the samples were thawed at room temperature. The thickness of cartilage was estimated using a digital caliper. The exact thicknesses were later confirmed by segmenting the surface of the cartilage and the cartilage-bone interphase from the PCD-CT images and calculating the distance in the mid part of the plug (caliper thickness error < 14%). This was then applied to the moduli calculations. The average thickness of the cartilage was 2.86 mm with a standard deviation of 0.62 mm. The osteochondral samples were glued to the bottom of a measuring chamber, which was then filled with PBS.

An indenter with a plane-ended head was driven into contact with the surface of the sample with a pre-stress of 12.5 kPa^1^. Contact was ensured with preconditioning by indenting the specimen five separate times, 2% of its thickness each time. The diameter of the indenter was 728 µm for one cadaver and 667 µm for the other due to a breakage of the first indenter. The change in indenter size was considered in the moduli calculations. A stress-relaxation protocol was implemented using a ramp strain rate of 100% per second with three compressive steps. Each step was 5% of the remaining uncompressed cartilage thickness with a relaxation period of 15 minutes after each step^2^.

The measurements were done with a custom-made testing system equipped with a high-precision load cell (resolution of 0.005 N, Sensotec, Columbus, OH, USA) and an actuator (resolution of 0.1 µm, PM500-1 A, Newport, Irvine, CA, USA). Equilibrium modulus was calculated as a linear fit of the stress-strain ratio at equilibrium points. Instantaneous modulus was calculated from the ramp phase of the third step. The moduli were calculated using the solution derived by Hayes *et al.*^3^ and the Poisson’s ratios used in the calculations were 0.2 and 0.5 for the equilibrium and instantaneous modulus, respectively^4^.

*Preprocessing and Reconstruction*

The PCD consisted of 24 smaller tiles with a one-pixel (100 µm) gap between the tiles. Tile gap interpolation utilizing mean filtering in the horizontal and vertical direction was applied to smooth out the tile edges for each LE, TE and HE bin images. Subsequently, images of all the energy bins were preprocessed using the signal-to-equivalent thickness correction^5^ (STC) and a ring artifact removal algorithm^6^. The former was used to correct beam hardening and the tilewise variations (Manuscript Figs. 4a and 4b)^5^. For STC correction, aluminum slabs with thicknesses of 0, 0.5, 0.95, 2.1, and 3.1 cm were imaged and the pixelwise fitting for all energy ranges was performed independently using the equation:

| $y_{i}\left( t_{i} \right)=O_{i}+A_{i}\cdot e^{a_{i}\cdot t_{i}},$ | (1) |
| --- | --- |

where *y_i_* is the observed counts for pixel *i*, *O_i_*, *A_i_*, and *a_i_* are the pixelwise fitting parameters, and *t_i_* is the aluminum thickness. Subsequently, the measured counts were transformed pixelwise to an equivalent aluminum thickness of the reference aluminum calibration material. This was performed separately for all the energy bin images.

To improve the signal-to-noise ratio, and to save memory on the reconstruction computer, two consecutive frames of the projection data were averaged yielding one projection per 0.5°. 3D reconstruction of each energy bin was calculated with the ASTRA Toolbox (toolbox for MATLAB, ver. 1.8) using the FDK method^7–9^. Finally, the image values were transformed from arbitrary attenuation units to Hounsfield units (HUs) (Manuscript Fig. 4c). All data analysis was done using MATLAB (R2018b, MathWorks, Natick, MA, USA). Only the LE and HE reconstruction images were used in the later analysis.

*Concentration Estimation for CA4+ and Gadoteridol*

Calibration curves were established using the solutions of known concentrations of the contrast agents. A first-degree polynomial equation was fitted to the measured calibration points between the known contrast agent concentrations and the measured HU values. Mass attenuation coefficients in units of HU·mL/mg were determined as the calibration curve slopes (Fig. S1a and S1c). The concentration estimation was validated with a validation series, using known mixtures of the contrast agents. These were used to investigate the accuracy of this calibration-based concentration estimation^10,11^ by comparing the calculated and true mixture concentrations (Fig. S1b and S1d).

The depthwise contrast agent concentrations within cartilage were calculated from the reconstructed images using the Beer-Lambert law and Bragg’s additive rule (Manuscript Figs. 4d and 4e), as in the earlier studies^10,11^:

| $\alpha_{E}=C_{I}\cdot\mu_{I,E}+C_{\mathrm{Gd}}\cdot\mu_{\mathrm{Gd},E},$ | (2) |
| --- | --- |

where *α_E_* is a HU value at energy bin *E*, *C*_I_ and *C*_Gd_ are the concentrations of iodine and gadolinium, respectively, and *µ*_I,_*_E_* and *µ*_Gd,_*_E_* are the measured mass attenuation coefficients for iodine and gadolinium at energy bin *E*. The used energy bins were LE and HE. By applying these energy bins to the equation (2), the CA4+ and gadoteridol concentrations can be solved as follows:

| $C_{I}=\frac{\alpha_{LE}\cdot\mu_{\mathrm{Gd},HE}-\alpha_{HE}\cdot\mu_{\mathrm{Gd},LE}}{\mu_{I,LE}\cdot\mu_{\mathrm{Gd},HE}-\mu_{I,HE}\cdot\mu_{\mathrm{Gd},LE}},$ | (3) |
| --- | --- |
| $C_{\mathrm{Gd}}=\frac{\alpha_{LE}\cdot\mu_{I,HE}-\alpha_{HE}\cdot\mu_{I,LE}}{\mu_{\mathrm{Gd},LE}\cdot\mu_{I,HE}-\mu_{\mathrm{Gd},HE}\cdot\mu_{I,LE}}.$ | (4) |

Subsequently, the contrast agent concentrations in the cartilage were transformed into contrast agent partitions by dividing the measured concentration with that of the surrounding contrast agent bath. The diffusion related decrease of the contrast agent concentrations in the surrounding bath was taken into consideration and calculated using the following equation:

| $C_{\mathrm{Bath}}=C_{\mathrm{Bath},0}-C_{\mathrm{AC}}\cdot\frac{V_{\mathrm{AC}}}{V_{\mathrm{Bath}}},$ | (5) |
| --- | --- |

where *C*_Bath_ is the concentration of the corresponding contrast agent in the bath, *C*_Bath,0_ is the initial concentration of the bath, *V*_Bath_ is the volume of the bath, *C*_AC_ is the concentration of the contrast agent in the articular cartilage at a given time, and *V*_AC_ is the volume of the cartilage estimated from the CT images.

The cartilage was segmented from the TE (30-100 keV) reconstructed images using Seg3D (ver. 2.4.3, CIBC, University of Utah, Salt Lake City, UT, USA). Cartilage surface, bone-cartilage interface, and a center point between these were recorded. The segmented cartilages were rotated so that the articular surface would be positioned on the axial plane, therefore, all the samples were oriented the same way. The volume-of-interest (VOI) for each sample was chosen based on the segmentation by selecting a point in the middle of the segmented surface of the cartilage and taking 20 pixels × 20 pixels (approximately 1.74 mm × 1.74 mm) square region-of-interest (ROI) around this point. This was used for all the depthwise slices of the cartilage (Manuscript Fig. 4f and 4g)^12^. After this, each ROI was averaged, and a depthwise profile was formed. All the profiles were interpolated to 100 points. The average cartilage thickness was 2.87 ± 0.63 mm, and this yielded approximately 33 ± 7 pixels for full cartilage thickness. Normalized CA4+ profile was acquired by dividing the CA4+ profile pointwise with the corresponding gadoteridol profile.

*Three-Material Decomposition Analysis*

We applied a three-material decomposition method, i.e. iodine, gadolinium, and bone, for one sample to test how well our concentration estimation method works near the subchondral bone. For this proof-of-concept evaluation, we adopted a method of Tao et al.^13^, which uses an additional constraints for estimation of the third basis material:

| $C_{Bone}=\rho_{Bone}\left( 1-\left( \frac{C_{I}}{\rho_{I}}+\frac{C_{Gd}}{\rho_{Gd}} \right) \right)\mathbf{,}$ | (6) |
| --- | --- |

where the *ρ_i_* denotes the mass density of the *i*^th^ basis material. We applied this proof-of-concept method for one cartilage sample, and interestingly we were able to demonstrate that the method could be used for differentiation between contrast enhanced deep zone and the calcified cartilage/bone (Fig. S2). The concentration estimation for CA4+ did start decreasing around the border of cartilage and calcified cartilage, i.e. from approx. 70 mg/mL to approx. 15 mg/mL, and the bone concentration estimation increased from approx. 197 mg/cm^3^ to approx. 199 mg/cm^3^.

*The Spatial Resolution and Modulation Transfer Function Analysis*

The spatial resolution of the imaging system was estimated by measuring the modulation transfer function (MTF) from a total energy slice images of the high contrast iodine tube (C = 72 I·mg/mL). The edge spread function (ESF) was determined from the high contrast target radially from five adjacent slices yielding a total of 40 profiles. The profiles were averaged, and the line spread function (LSF) was estimated by taking a derivate of the ESF. Finally, the upsampled MTF was estimated from the normalized Fourier transform magnitude of the zero-padded LSF profile. The MTF50% and MTF10% values for the PCD-CT system were 2.43 1/mm and 4.27 1/mm, respectively (Fig. S3).

**References**

1. Korhonen, R. K. *et al.* Fibril reinforced poroelastic model predicts specifically mechanical behavior of normal, proteoglycan depleted and collagen degraded articular cartilage. *J. Biomech.* **36**, 1373–1379 (2003).

2. Prakash, M. *et al.* Near-infrared spectroscopy enables quantitative evaluation of human cartilage biomechanical properties during arthroscopy. *Osteoarthr. Cartil.* **27**, 1235–1243 (2019).

3. Hayes, W. C., Keer, L. M., Herrmann, G. & Mockros, L. F. A mathematical analysis for indentation tests of articular cartilage. *J. Biomech.* **5**, 541–551 (1972).

4. Kiviranta, P. *et al.* Collagen network primarily controls Poisson’s ratio of bovine articular cartilage in compression. *J. Orthop. Res.* **24**, 690–699 (2006).

5. Jakubek, J., Vavrik, D., Pospisil, S. & Uher, J. Quality of X-ray transmission radiography based on single photon counting pixel device. *Nucl. Instruments Methods Phys. Res. Sect. A Accel. Spectrometers, Detect. Assoc. Equip.* **546**, 113–117 (2005).

6. Münch, B., Trtik, P., Marone, F. & Stampanoni, M. Stripe and ring artifact removal with combined wavelet — Fourier filtering. *Opt. Express* **17**, 1844–1856 (2009).

7. van Aarle, W. *et al.* The ASTRA Toolbox: A platform for advanced algorithm development in electron tomography. *Ultramicroscopy* **157**, 35–47 (2015).

8. van Aarle, W. *et al.* Fast and flexible X-ray tomography using the ASTRA toolbox. *Opt. Express* **24**, 25129 (2016).

9. Palenstijn, W. J., Batenburg, K. J. & Sijbers, J. Performance improvements for iterative electron tomography reconstruction using graphics processing units (GPUs). *J. Struct. Biol.* **176**, 250–253 (2011).

10. Bhattarai, A. *et al.* Quantitative Dual Contrast CT Technique for Evaluation of Articular Cartilage Properties. *Ann. Biomed. Eng.* **46**, 1038–1046 (2018).

11. Honkanen, M. K. M. *et al.* Imaging of Proteoglycan and Water Contents in Human Articular Cartilage with Full-Body CT Using Dual Contrast Technique. *J. Orthop. Res.* **37**, 1059–1070 (2019).

12. Saukko, A. E. A. *et al.* Dual Contrast CT Method Enables Diagnostics of Cartilage Injuries and Degeneration Using a Single CT Image. *Ann. Biomed. Eng.* **45**, 2857–2866 (2017).

13. Tao, S., Rajendran, K., McCollough, C. H. & Leng, S. Material decomposition with prior knowledge aware iterative denoising (MD-PKAID). *Phys. Med. Biol.* **63**, 195003 (2018).


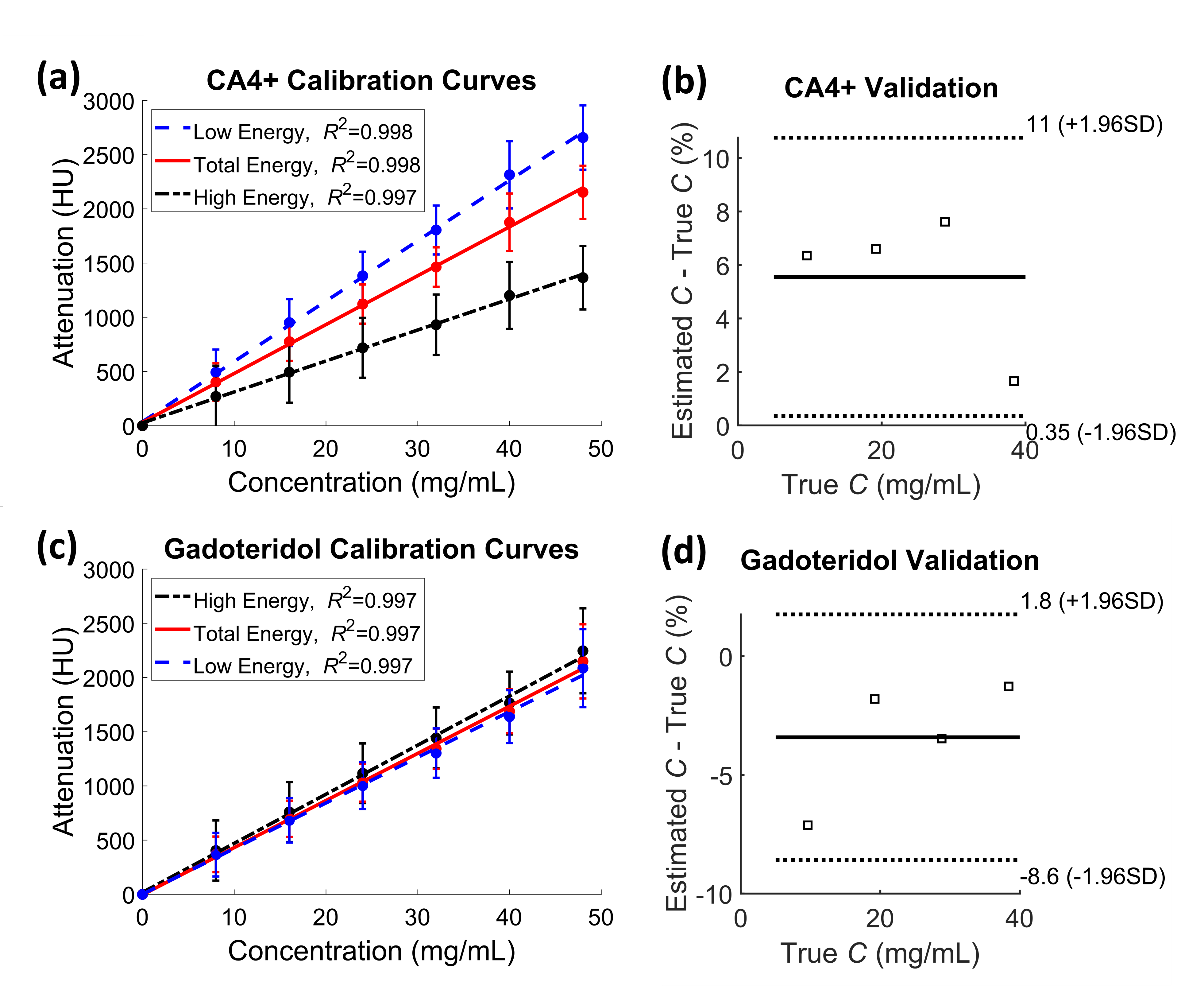


**SUPPLEMENTARY FIGURE S1. CA4+ and gadoteridol calibration curves (a) and (c), respectively, used for the concentration estimation. The error bars in sub-figures (a) and (c) indicates the standard deviation. Error between the measured and the true concentration C of the contrast agents for (b) CA4+ and (d) gadoteridol, respectively, visualized using Bland-Altman plot. The difference in percent is calculated by dividing the concentration difference with true concentration.**


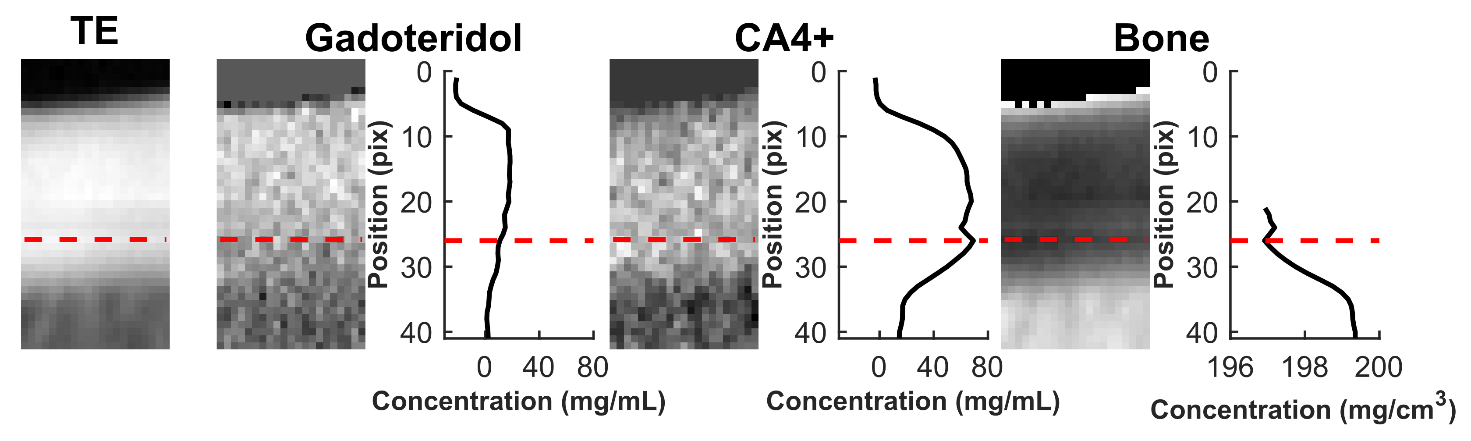


**SUPPLEMENTARY FIGURE S2. Proof-of-concept image of the three-material decomposition. On the left there is total energy (TE) image for refence. For each of the three materials, there are concentration map images and profiles. The articular cartilage surface can be seen in the upper part of the images and the bone is in the lower part of the images. The dotted red line indicates roughly where the cartilage ends, and the calcified cartilage starts.**


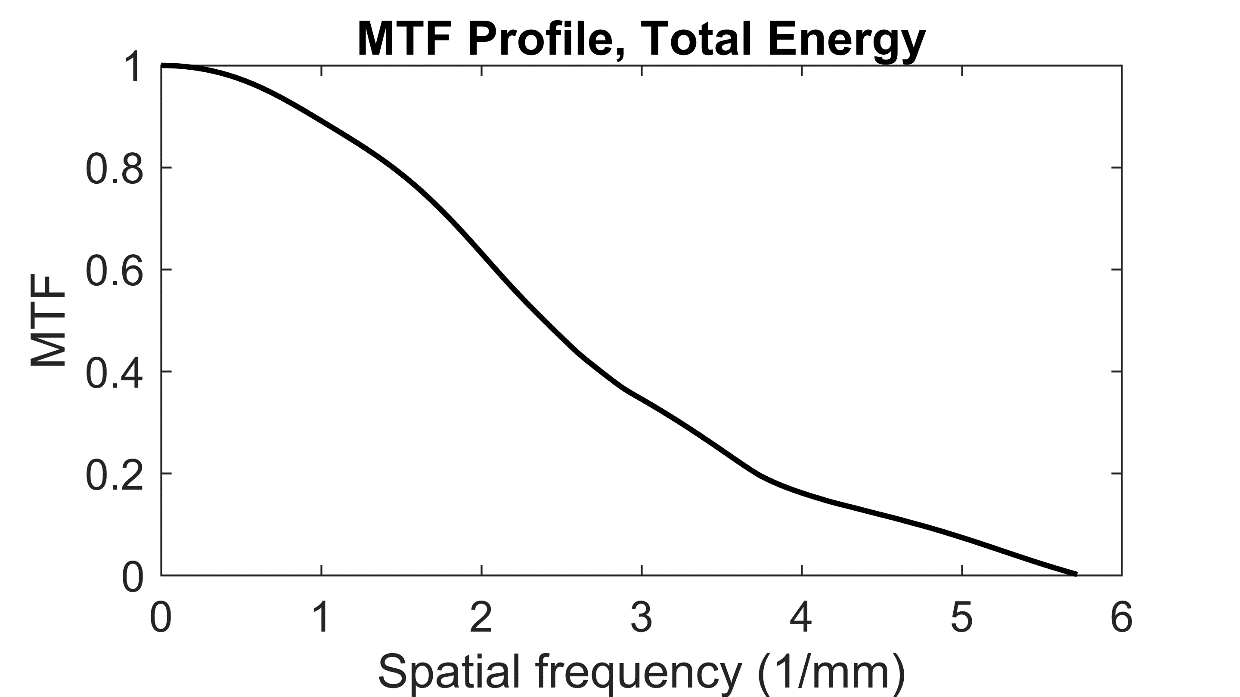


**SUPPLEMENTARY FIGURE S3.** **The modulation transfer function (MTF) profile was used to estimate the spatial resolution of the imaging system** **MTF was determined from a total energy image of a high contrast iodine tube. Total of 40 locations from five adjacent slices were used in the analysis. The MTF50% and MTF10% values were 2.43 1/mm and 4.27 1/mm, respectively.**
